# Ewing Sarcoma in the Pediatric Population: Predictors of Survival Within the United States

**DOI:** 10.5435/JAAOSGlobal-D-24-00281

**Published:** 2024-10-21

**Authors:** Matthew E. Wells, Michael D. Eckhoff, William Davis, Vishwajeet Singh, Rajiv Rajani, Elizabeth M. Polfer

**Affiliations:** From the Department of Orthopedic Surgery, William Beaumont Army Medical Center (Dr. Wells, Dr. Eckhoff, and Dr. Polfer); the Department of Orthopedic Surgery, Texas Tech University Health Sciences Center El Paso (Dr. Wells, Dr. Eckhoff, Dr. Davis, Dr. Rajani, and Dr. Polfer); and Biostatistics and Epidemiology Consulting Lab, Texas Tech University Health Sciences Center El Paso, El Paso, TX (Dr. Singh).

## Abstract

**Introduction:**

Bone and joint tumors are the third most common cause of pediatric cancer–related deaths in the United States. Although there have been improvements in survival rates among pediatric cancer patients over the past few decades, bone and joint cancers remain the exception. Considering current clinical trials involving novel targeted therapies, the establishment of updated mortality rates and predictors of survival for this cancer would be prudent. This investigation sought to determine updated 5-year survival rates and predictors of survival among pediatric Ewing sarcoma (ES) of bone treated within the United States.

**Methods:**

The National Cancer Database was retrospectively inquired for all pediatric ES cases within the most updated bone and joint public use file available in September 2022. The reported data were truncated to only include patients with reported 5-year vital (ie, survival) status. Cox proportional hazard regression was conducted on both the truncated data and the entire cohort to validate the findings. The patients were then separated into alive versus deceased cohorts, and univariate regression analysis was done followed by multivariable regression of notable variables of interest.

**Results:**

Overall, an aggregated 5-year survival rate of 74.5% was found in the included patient cohort. Patients with localized cancer had a comparatively improved 5-year survival rate of 84.70% as opposed to those with macrometastatic disease on presentation with a survival rate of 50.4%. Patient demographic-, tumor-, and treatment-specific variables all demonstrated an effect on survival. The multivariable predictors of worse mortality were found to include older age, larger tumor size (>8 cm), macrometastatic disease on presentation, and positive surgical margins.

**Conclusion:**

This analysis serves to establish updated survival rates of pediatric ES treated within the United States to set standards for comparison among future studies. Continued multi-institutional and international collaboration is needed to optimize current treatment results and develop novel targeted therapies.

Bone sarcomas are the third leading cause of pediatric cancer-related deaths in the United States.^1^ Although there have been improvements in survival rates among pediatric cancer patients over the past 30 years, bone sarcomas, unfortunately, remain the exception.^2-5^ Despite considerable advances in characterizing molecular and genetic predispositions for these cancers,^6-8^ survival rates have plateaued overall.^9,10^ Ewing sarcoma (ES) remains the second most common primary bone sarcoma in adolescents, second to osteosarcoma, and serves as a predominant causality for the stagnant survival rates.^10^

ES was originally characterized as a round cell neoplasm of bone and later as a subset of primitive neuroectodermal tumors.^11-13^ The World Health Organization's most recent update defines these tumors as “Ewing sarcoma” with the pathognomonic gene fusions involving the fused in sarcoma and E26 transformation-specific gene families, most classically *EWSR1-FLI1*, with rare variants containing *STAG2*, *CDKN2A*, or *TP53* mutations.^14^ ES displays a single peak incidence of around 15 years.^15^ Treatment typically includes neoadjuvant chemotherapy, wide resection with limb salvage, and adjuvant chemotherapy. However, additional radiation treatment may be employed in select cases or as a means of definitive control in lieu of surgical resection with irresectable tumors.^16-18^ Based on previous epidemiologic reports, patients with localized disease demonstrate 5-year survival rates of approximately 72%, whereas those initially presenting with metastases demonstrate 5-year survival rates of approximately 28%.^19-22^

Since the initial prognostic improvement with the introduction of systemic chemotherapy in the 1970s, ES survival rates have remained bleak, particularly for those with metastatic and recurrent disease.^17,23-25^ Despite an improved understanding of the pathogenesis and altered treatment protocols of ES,^6^ survival rates overall among this cancer have remained relatively stagnant. Considering clinical trials involving novel targeted therapies, the establishment of updated survival rates and predictors of mortality for pediatric ES patients would be prudent. This investigation sought to determine the most updated 5-year survival rates among these pediatric patients diagnosed with ES and treated within the United States. Secondary outcomes included multivariable determination of patient, tumor-, and treatment-specific effects on survival rates.

## Methods

The National Cancer Database (NCDB) was inquired for all pediatric ES cases within the most updated bone and joint public use file available in September 2022 (patients reported from 2008 to 2017). The NCDB contains deidentified data, and therefore was exempted from formal institutional board review as nonhuman subject research. This database includes 34 million patient records with a 90% rate of 5-year follow-up,^26^ capturing approximately 70% of all patients newly diagnosed with cancer.^27,28^ Reported pediatric (<18 years of age) sarcomas were extracted using appropriate International Classification Diseases for Oncology coding. The International Classification Diseases for Oncology codes 9260/3 and 9264/3 were used for the identification and separation of ES for subsequent analysis based on reported histologic diagnostic confirmation. Primary tumor locations were narrowed and organized to specific areas of interest, including the face and skull, axial skeleton (spine, ribs, manubrium), pelvis, and extremities for clinical in concordance with the American Joint Committee on Cancer (AJCC) classification (Table 1). Patients were then separated into alive versus deceased cohorts based on reported 5-year vital (i.e. survival) status. Chi-square test or Fisher exact test was used to determine whether an association exists among the categorical variables of interest in these patient cohorts (Table 2). Continuous data were compared between alive versus deceased by *t*-test or Wilcoxon rank sum test. To avoid violating the proportional hazards assumption, the data thereafter required truncation to the first 10 years reported (which allowed for a minimum 5-year follow-up) for relevant survival analysis. A Cox proportional hazard regression was conducted on both the truncated data and the entire cohort to validate the findings. Hazard ratios (HRs), 95% confidence intervals (CIs), and *P* values were used to describe the Cox model. Test for proportional hazard assumption after fitting the Cox model was explored based on Schoenfeld residuals. After a univariate analysis was completed (Table 3), a backward stepwise procedure was used to select the optimal set of variables for multivariable (Figure 2) regression models. Significance was considered at *P* < 0.05. All statistical tests were conducted using STATA (V17).

**Table 1:** Distribution of Overall Patient-, Tumor-, and Treatment-specific Characteristics for Reported Pediatric Ewing Sarcoma Patients

Factor	Value
N	1996
Age at diagnosis (yrs), mean (SD)	11.6 (4.2)
Age groups (yrs)	
0-5	206 (10.32%)
6-11	633 (31.71%)
12-17	1157 (57.97%)
Sex	
Male	1160 (58.12%)
Female	836 (41.88%)
Race	
White	1767 (88.53%)
African American/Black	59 (2.96%)
Other	170 (8.52%)
Ethnicity	
Non-Hispanic	1611 (84.79%)
Hispanic	289 (15.21%)
Insurance status	
Not insured	50 (2.58%)
Private insurance/managed care	1341 (69.23%)
Government	546 (28.19%)
Primary tumor location	
Lower extremity	629 (32.62%)
Upper extremity	243 (12.60%)
Facial & skull + mandible-specific	128 (6.64%)
Spine + axial skeleton	395 (20.49%)
Pelvis	493 (25.57%)
Extremity, unspecified + unspecified bone	40 (2.07%)
Tumor size	
≤8 cm	329 (16.48%)
>8 cm	193 (9.67%)
Primary tumors could not be assessed	873 (43.74%)
Missing	601 (30.11%)
Margin status	
Negative	777 (38.93%)
Positive	156 (7.82%)
No primary site surgery	859 (43.04%)
Not reported	204 (10.22%)
Metastasis status	
No	1727 (86.52%)
Yes	241 (12.07%)
Not reported	28 (1.40%)
Metastasis—bone	
No	714 (35.77%)
Yes	103 (5.16%)
Unknown/missing	1179 (59.07%)
Metastasis—lung	
No	639 (32.01%)
Yes	178 (8.92%)
Unknown/missing	1179 (59.07%)
Surgical procedure of the primary site	
Wide resection (with limb salvage)	671 (34.32%)
Local excision	332 (16.98%)
No surgery performed	859 (43.94%)
Amputation	93 (4.76%)
Surgery	
No	859 (43.27%)
Yes	1126 (56.73%)
Chemotherapy	
No	79 (4.01%)
Yes	1892 (95.99%)
Radiation	
No	1571 (79.50%)
Yes	405 (20.50%)
Treatment type	
Chemotherapy and surgery	1103 (55.26%)
Surgery without chemotherapy	23 (1.15%)
Chemotherapy without surgery	789 (39.53%)
No chemotherapy or surgery	81 (4.06%)
Mortality (5 year)	
Alive	1361 (73.41%)
Deceased	493 (26.59%)

**Table 2:** Distribution of Characteristics by Mortality Status at 5-Year Follow-up Among Ewing Sarcoma Patients

Factor	Alive	Deceased	*P*
N	1361	493	
Age at diagnosis, mean (SD)	11.3 (4.3)	12.5 (3.8)	<0.001
Last contact or death, months from Dx, median (IQR)	68.8 (38.8, 107.3)	27.8 (15.9, 44.9)	<0.001
Age—categorized			<0.001
0-5	163 (11.98%)	27 (5.48%)	
6-11	465 (34.17%)	131 (26.57%)	
12-17	733 (53.86%)	335 (67.95%)	
Sex			0.96
Male	789 (57.97%)	285 (57.81%)	
Female	572 (42.03%)	208 (42.19%)	
Race			0.75
White	1204 (88.46%)	441 (89.45%)	
African American/Black	40 (2.94%)	15 (3.04%)	
Other	117 (8.60%)	37 (7.51%)	
Hispanic status			0.06
Non-Hispanic	1110 (85.52%)	383 (81.84%)	
Hispanic	188 (14.48%)	85 (18.16%)	
Insurance status			0.37
Not insured	34 (2.57%)	15 (3.17%)	
Private insurance/managed care	927 (69.96%)	315 (66.60%)	
Government	364 (27.47%)	143 (30.23%)	
Primary tumor locations			<0.001
Lower extremity	454 (34.37%)	138 (29.30%)	
Upper extremity	176 (13.32%)	46 (9.77%)	
Facial and skull + mandible specific	101 (7.65%)	14 (2.97%)	
Spine + axial skeleton	279 (21.12%)	89 (18.90%)	
Pelvis	282 (21.35%)	176 (37.37%)	
Extremity, unspecified + unspecified bone	29 (2.20%)	8 (1.70%)	
Tumor size			<0.001
≤8 cm	253 (18.59%)	40 (8.11%)	
>8 cm	121 (8.89%)	58 (11.76%)	
Primary tumors cannot be assessed	562 (41.29%)	290 (58.82%)	
Missing	425 (31.23%)	105 (21.30%)	
Metastasis status			<0.001
No	1203 (88.39%)	382 (77.48%)	
Yes	139 (10.21%)	102 (20.69%)	
Unknown/NA	19 (1.40%)	9 (1.83%)	
Metastasis—bone			<0.001
No	589 (43.28%)	125 (25.35%)	
Yes	41 (3.01%)	62 (12.58%)	
Unknown/missing	731 (53.71%)	306 (62.07%)	
Metastasis—lung			<0.001
No	518 (38.06%)	121 (24.54%)	
Yes	112 (8.23%)	66 (13.39%)	
Unknown/missing	731 (53.71%)	306 (62.07%)	
Margin status			<0.001
Negative	600 (44.09%)	119 (24.14%)	
Positive	109 (8.01%)	38 (7.71%)	
No primary site surgery	509 (37.40%)	291 (59.03%)	
Unknown/missing	143 (10.51%)	45 (9.13%)	
Surgical procedure of the primary site			<0.001
Wide resection (with limb salvage)	510 (38.23%)	111 (22.98%)	
Local excision	248 (18.59%)	62 (12.84%)	
No surgery performed	509 (38.16%)	291 (60.25%)	
Amputation	67 (5.02%)	19 (3.93%)	
Surgery			<0.001
No	509 (37.59%)	291 (59.39%)	
Yes	845 (62.41%)	199 (40.61%)	
Chemotherapy			0.50
No	51 (3.80%)	22 (4.53%)	
Yes	1292 (96.20%)	464 (95.47%)	
Radiation			0.39
No	1065 (79.06%)	397 (81.02%)	
Yes	282 (20.94%)	93 (18.98%)	
Treatment type			<0.001
Chemotherapy and surgery	827 (60.76%)	194 (39.35%)	
Surgery without chemotherapy	18 (1.32%)	5 (1.01%)	
Chemotherapy without surgery	465 (34.17%)	270 (54.77%)	
No chemotherapy or surgery	51 (3.75%)	24 (4.87%)	

NA = not available

**Table 3:** Unadjusted Associations of Factors With Survival Status Among Ewing sarcoma Patients

Factors	HR	95% CI	*P*
Age groups, yr			
0-5	1	—	—
6-11	1.43	0.94-2.16	0.09
12-17	2.27	1.54-3.37	<0.001
Sex			
Male	1	—	—
Female	1.02	0.85-1.22	0.82
Race			
White	1	—	—
African American/Black	1.14	0.68-1.91	0.61
Other	0.95	0.68-1.33	0.77
Ethnicity			
Non-Hispanic	1	—	—
Hispanic	1.33	1.05-1.68	0.02
Insurance			
Not insured	1	—	—
Private insurance/managed care	0.79	0.47-1.36	0.40
Government	0.91	0.53-1.58	0.75
Primary tumor locations			
Lower extremity	1	—	—
Upper extremity	0.88	0.63-1.22	0.44
Facial and skull + mandible specific	0.53	0.30-0.92	0.02
Spine + axial skeleton	1.08	0.83-1.41	0.58
Pelvis	1.96	1.57-2.46	<0.001
Extremity, unspecified + unspecified bone	1.03	0.51-2.10	0.93
Tumor size			
≤8 cm	1	—	—
>8 cm	2.51	1.68-3.75	<0.001
Primary tumors cannot be assessed	2.63	1.89-3.66	<0.001
Missing	1.63	1.13-2.34	0.01
Metastasis status			
No	1	—	—
Yes	2.33	1.87-2.91	<0.001
Unknown/NA	2.03	1.05-3.94	0.04
Bone metastasis			
No	1	—	—
Yes	5.19	3.82-7.04	<0.001
Unknown/missing	1.58	1.28-1.96	<0.001
Lung metastasis			
No	—	—	—
Yes	2.28	1.69-3.07	<0.001
Unknown/missing	1.45	1.17-1.80	0.001
Margin status			
Negative	1	—	—
Positive	1.71	1.19-2.47	0.004
No primary site surgery	2.89	2.34-3.58	<0.001
Unknown/missing	1.66	1.18-2.34	0.004
Surgical procedure of the primary site			
Wide resection (with limb salvage)	1	—	—
Local excision	1.19	0.87-1.62	0.27
No surgery performed	2.66	2.13-3.30	<0.001
Amputation	1.28	0.79-2.09	0.32
Surgery			
Yes	1	—	—
No	2.43	2.03-2.91	<0.001
Chemotherapy			
Yes	1	—	—
No	1.86	1.21-2.85	0.01
Radiation			
No	1	—	—
Yes	0.88	0.70-1.10	0.28
Treatment type			
Chemotherapy and surgery	1	—	—
Surgery without chemotherapy	1.56	0.64-3.79	0.33
Chemotherapy without surgery	2.39	1.99-2.88	<0.001
No chemotherapy or surgery	3.28	2.13-5.05	<0.001

CI = confidence interval, HR = hazard ratio, NA = not available

## Results

Overall, 1996 pediatric ES patients were identified within the selected bone and joint public use file, which was truncated to 1854 patients to ensure a reported minimum 5-year follow-up.

### Five-Year Survival

**Figure 1:**
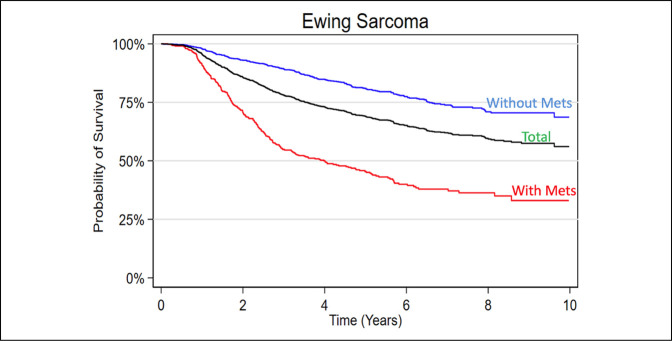
Graph showing Kaplan-Meier 10-year survival analysis of the included Ewing sarcoma patient cohort.

Overall, an aggregated 5-year survival rate of 73.92% was found in the entire cohort. Patients with localized cancer had a comparatively improved 5-year survival rate of 84.70% as opposed to those with macrometastatic disease on presentation, with a survival rate of 50.38% (Figure 1).

### Patient-Specific Prognostic Factors

**Figure 2:**
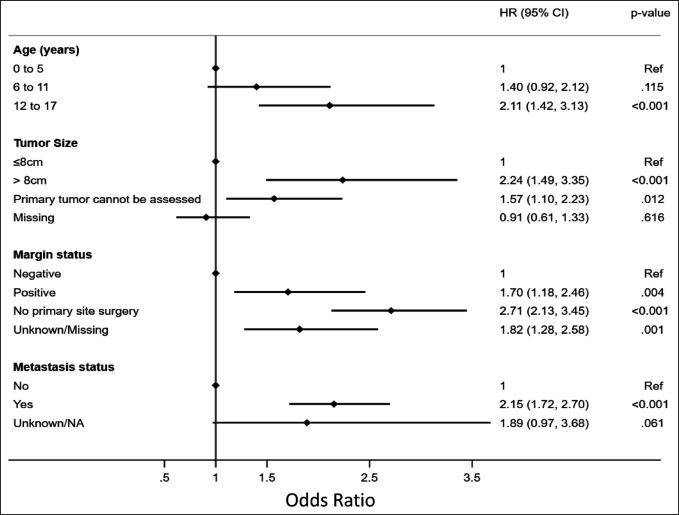
Graph/plot showing multivariable analysis of independent variables predictive of survival in pediatric patients diagnosed with Ewing sarcoma.

Most patients were of adolescent age (57.6%) with the majority being non-Hispanic, White males (Table 1). Most patients had private insurance or other form of managed care (69.23%) followed by government-based insurance (28.19%; Table 1). When separated into alive vs deceased groups, adolescent age at diagnosis was proportionally more common in the deceased group (67.95% vs. 53.86), whereas no notable differences were observed between sex, race, Hispanic status, or insurance type observed (Table 2). Unadjusted patient demographic-specific variables associated with worse survival rates included increasing age (12 to 17 years, HR [95% CI] = 2.27 [1.54 to 3.37]) and Hispanic ethnicity (HR [95% CI] = 1.33 [1.05 to 1.68]), whereas sex, overall race, and insurance status had no notable effect (Table 3). After multivariable analysis, only adolescent age (12 to 17, HR [95% CI] = 2.11 [1.42 to 3.13]) was considered a notable predictor of worse 5-year survival (Figure 2).

### Tumor-Specific Prognostic Factors

The most common primary tumor locations were reported within the lower extremities (32.62%) or pelvis (25.57%; Table 1). Approximately 12% of patients were found to have clinically identified macrometastatic disease on presentation. Of the limited cases with the reported largest tumor dimension, most were tumor sizes of <8 cm (AJCC T1 classification). When separated into alive vs. deceased groups, tumor-specific characteristics more commonly reported in the deceased group included pelvic tumor location (37.37% vs. 21.35), larger tumor size (>8 cm: 11.76% vs. 8.89%), and metastasis on presentation (20.69% vs. 10.21%; Table 2). Unadjusted tumor-specific variables associated with worse survival rates included primary tumor location (pelvis, HR [95% CI] = 1.96 [1.57 to 2.46]), larger tumor size (>8 cm, HR [95% CI] = 2.51 [1.68 to 3.75]), and presence of metastasis at the time of diagnosis (HR [95% CI] = 2.33 [1.87 to 2.91]; Table 3), whereas grade had no notable effect. Primary tumor locations involving facial and skull or mandible-specific locations had lower mortality rates. After multivariable analysis, larger tumor size (>8 cm, HR [95% CI] = 2.24 [1.49 to 3.35]) and positive metastases on presentation (HR [95% CI] = 2.15 [1.72 to 2.70]) were associated with considerable significance in predicting worse 5-year survival (Figure 2).

### Treatment-Specific Prognostic Factors

Most patients underwent treatment with a sequenced chemotherapy and surgical resection; approximately 20% of patients had received some degree of radiation treatment (Figure 1). When separated into alive vs deceased groups, treatment-specific characteristics more commonly reported in the deceased group included no primary surgery being performed, whereas no differences were observed in chemotherapy alone or radiation alone treatments (Table 2). Alive patients were reported to undergo combined chemotherapy and surgery at a markedly higher rate than deceased patients (60.76% versus 39.35%, respectively), who more often underwent nonsurgical management. Unadjusted treatment-specific variables associated with worse survival rates were positive margins after surgical resection (HR [95% CI] = 1.71 [1.19 to 2.47]), tumors in which no primary site surgery was performed (HR [95% CI] = 2.66 [2.13 to 3.30]), and patients treated without chemotherapy or surgery (HR [95% CI] = 3.28 [2.13 to 5.05]; Table 3). After multivariable analysis, only positive margin status (HR [95% CI] = 1.70 [1.18 to 2.46]) was considered significant in predicting worse 5-year survival (Figure 2).

## Discussion

ES remains the second most common pediatric primary malignant bone tumor reported in the United States. Although the advent of combined chemotherapy and wide resection protocols have markedly improved outcomes, novel panacea treatments remain elusive. This study demonstrated a 74.5% overall 5-year survival rate, with improved survival in localized tumors (84.7%) compared with patients presenting with metastatic disease (50.4%). Collectively, these results imply optimistically improving short-midterm survival, particularly for patients presenting with metastases. Patient demographic-, tumor-, and treatment-specific variables demonstrated an effect on survival. The multivariable predictors of worse mortality were found to include older age, larger tumor size (>8 cm), macrometastatic disease on presentation, and positive surgical margins.

### Survival Rates

Overall, an aggregated 5-year survival rate of 74.5% was found among all included patients. Patients with localized disease had a comparatively improved 5-year survival rate of 84.70% as opposed to patients with macrometastatic disease on presentation with a survival rate of 50.4% (Figure 1). These findings are consistent, albeit slightly higher, compared with similar epidemiology-based and review studies for localized (approximately 70% to 80%) and metastatic (approximately 30% to 40%) disease.^17,20,29-33^ However, many of these larger population-driven and hospital database–driven reports are 1 to 2 decades old. Therefore, the authors believe that the improved survival rates reported in this study are likely a result of gradual improvement/emphasis on multidisciplinary care provided at larger tertiary referral centers with greater access to randomized control trials.^34-37^

### Patient-Specific Prognostic Factors

Increasing age was associated with overall worse survival rates, with adolescents demonstrating worse 5-year survival rates compared with younger age groups. This is consistent with previous findings that have shown the survival rate of ES to be inversely proportional to age among pediatric and young adult populations.^38-40^ Ewing tumor size and location have some purported age dependence with adolescents having markedly larger tumors and higher incidence of occurrence within the pelvis and axial skeleton as compared with preadolescent patients.^41^ Given the worse prognosis associated with axial tumors,^42^ this was likely a contributor to worse outcomes with increasing age in this analysis.

Diagnosis of ES primarily occurred in non-Hispanic White males, which is consistent with ES's historical predilection for European ancestries and strikingly low incidence in populations of African descent.^15^ This is likely secondary to genetic germline variants that are protective for African Americans.^43^ However, there is a tumor dedifferentiation risk of ES in people of African descent, which may be a result of a specific genetic alteration within the EGR2 gene.^44^ Regardless, patient sex and race did not account for notable differences in mortality status.

### Tumor-Specific Prognostic Factors

As expected, larger tumor size and metastatic disease on presentation were both associated with worse overall survival rates. These findings are consistent with tumor staging through the AJCC updated staging guidelines for primary bone tumors.^45^ Importantly, the most updated AJCC staging system (eighth edition) separates pelvis and spinal primary bone tumors to better reflect the overall difference in survival or treatments, respectively, as compared with appendicular tumors.^42,45^ It should also be noted that this study's analysis showed notable differences in tumor location among deceased and nondeceased patients diagnosed with ES, with deceased patients having notably higher percentages of pelvis locations (Tables 2 and 3). Axial primary bone malignancies, particularly within the pelvis, have been convincingly proven to have worse long-term survival rates compared with appendicular tumors,^46-53^ hence the updates to the AJCC staging system. It is uncertain as to why pelvic tumor location did not remain a notable predictor of worse mortality after the adjusted multivariable analysis (Table 3 versus Figure 2); however, this may reflect improvements in targeted radiation and chemotherapy treatments. Overall, improvements in targeted adjuvant treatments and the emphasis on multidisciplinary care are likely the two drivers behind the improved metastatic disease and/or pelvic tumor location survival rates.

The updated AJCC also lumps the appendicular skeleton with trunk, skull, and facial bone tumors for classification purposes of primary bone tumors. This study found that facial and mandible-specific primary Ewing tumor locations have markedly improved survival compared with appendicular tumors. These facial and mandible-specific primary tumors may be more easily noticed by patients and parents and therefore diagnosed earlier and treated in a timelier manner. However, these findings appear to contrast with similar survival rates to contemporary appendicular tumors.^54-56^ Although unclear for the causality, facial and mandible-specific tumors may need to be considered for separate categorization in newer editions of the AJCC Cancer Staging Manual.

### Treatment-Specific Prognostic Factors

Before the 1970s, amputation was the only believed methodology for managing local tumor burden.^57^ The shift to combined chemotherapy with limb-preservation surgery revolutionized outcomes and patient functionality.^58,59^ As expected, mortality was found to be markedly affected by treatment type, with chemotherapy and surgery combination treatment associated with improved 5-year survival rates in this analysis. Of note, limb-preservation surgery is contingent on the tumor's candidacy for resection and the patient/parents' desire for that surgery. For example, large and invasive pelvic tumors or metastatic burdens may preclude patients from undergoing combined chemotherapy and surgical management. These patients may be managed with isolated radiation treatments or combined with chemotherapy for palliative treatment. In this investigation, ES patients treated with likely isolated palliative radiation showed a trend toward improved survival compared with no treatment. We believe that this was likely unresectable tumors in central locations such as the previously mentioned pelvic location subgroup.

Among patients who undergo tumor resection, survival rates have been predicated on margin status and tumor response to chemotherapy. Similarly, this study showed that positive margins after surgical resection were associated with lower 5-year survival rates. Unfortunately, there is considerable variability in the methodology of reporting margins.^60^ For example, standard teaching stressed the importance of a 3-cm bony margin and soft-tissue layer surrounding the tumor.^61^ However, less stringent 1.5-cm margins have demonstrated equivalent oncologic outcomes in osteosarcoma.^62^ Despite these controversies, the basic principle of reducing tumor burden and local recurrence through negative margins upon wide resection persists.^19,50,63,64^ Histologic response to chemotherapy is important in ES^46^; however, this variable is not reported in the NCDB. Providers should bear in mind that even with successful treatment and remission past the 5-year mark, patients may require continued monitoring from their regular oncologist with referral to the musculoskeletal oncology surgeon as needed. Even with curative treatment without recurrence, there are notable short- and long-term treatment-related complications as a result of the chemotherapy and resection of these malignant bone tumors.^65,66^

### Limitations

There are several limitations to the use of large databases. Although the NCDB captures most new cancer patients in the United States^27,28^ and displays a high rate of 5-year follow-up,^26^ it is ultimately a hospital-based data set and not population based (such as Surveillance, Epidemiology, and End Results data). Therefore, patient inclusion is specific only to patients treated by hospitals accredited by the American College of Surgeons Committee on Cancer. Furthermore, analysis is limited to variables provided, which often results in the lack of specific data granularity. This includes exact tumor location, lack of *EWSR1* status for diagnostic confirmation, histologic response to chemotherapy, radiation dosing, type of chemotherapy received, or patients' involvement in clinical trials. The NCDB seeks to be a comprehensive database consistent in reporting variables among tumors. However, this may lead to errors in reporting or erroneous variables such as reporting various grades of ES of bone, which is generally universally considered undifferentiated.

In addition, the NCDB is unable to link separate or associated cancer diagnoses for the same patient, which may serve pertinent in patients with genetic predispositions. It reports each individual cancer diagnosis as a different case. However, researchers can exclude patients indicated to have a history of other cancers, thereby minimizing the effect of prior malignant neoplasms, as was done in this analysis. Furthermore, we were unable to report on survival rates regarding patients who initially presented with localized disease with later progression to metastatic disease despite having received treatment. Finally, this analysis was only able to report overall survival rather than event-free survival rates as recurrence and specific complications are not explicitly provided by the NCDB.

### Looking Forward—Treatments on the Horizon

Compressed chemotherapy induction with vincristine, doxorubicin, and cyclophosphamide remains the standard for ES within the United States.^67,68^ Although various trials continue, major changes in chemotherapy toward either malignancy have not been implemented in the United States in a decade. Current efforts in novel treatments include investigations into stereotactic radiosurgery, proton beam therapy, and even some targeted immunotherapies.^69^ Low incidence rates and difficulty obtaining adequately powered clinical trials remain burdensome for these investigations.

## Conclusion

ES remains the second most common primary bone tumor in pediatric and adolescent patients in the United States with reportedly stagnant survival rates across the past few decades. Overall, this study's ES cohort exhibited an 84.7% 5-year survival rate in patients with localized disease compared with 50.4% for patients presenting with metastatic disease (73.9% overall). The improved survival for metastatic patients compared with prior studies is likely a reflection of improvements in adjuvant treatments and the establishment of multidisciplinary care teams. Multivariable regression showed that older age, larger tumor size, macrometastases, and positive margins all result in worse 5-year survival rates. This analysis serves to establish updated survival rates of pediatric ES treated within the United States to set standards for comparison among future studies. Continued multi-institutional and international collaboration is needed to optimize current treatment results and develop novel targeted therapies.
